# Cl©Li_5_Cl_5_^−^: A Star-like Superhalogen Anion Featuring a Planar Pentacoordinate Chlorine at the Center

**DOI:** 10.3390/molecules29163831

**Published:** 2024-08-12

**Authors:** Li-Xia Bai, Cai-Yue Gao, Jin-Chang Guo, Si-Dian Li

**Affiliations:** Key Laboratory of Materials for Energy Conversion and Storage of Shanxi Province, Institute of Molecular Science, Shanxi University, Taiyuan 030006, China; bailixia2021@163.com (L.-X.B.); gaocaiyue@sxu.edu.cn (C.-Y.G.)

**Keywords:** planar pentacoordinate chlorine, superhalogen anion, global minimum, multicenter bonding, aromaticity

## Abstract

Among the known planar pentacoordinate atoms, chlorine is missing due to its large radius and high electronegativity. Herein, we report the first star-like superhalogen anion *D*_5*h*_ Cl©Li_5_Cl_5_^−^ (**1**), which contains a planar pentacoordinate chlorine (ppCl) at the center. Computer structural searches and high-level calculations reveal that **1** is a true global minimum (GM) on the potential energy surfaces. Molecular dynamics simulations indicate it is kinetically stable against isomerization or decomposition. Although detailed chemical bonding analyses reveal one delocalized 6c-2e σ bond over the Cl©Li_5_ central unit and five delocalized 3c-2e σ bonds along the periphery, while aromaticity has very little beneficial effect on stability, instead, ionic interaction dominates the stability of the system. More encouragingly, with the large HOMO–LUMO energy gap of 7.66 eV and vertical detachment energy of 7.87 eV, the highly chemically inert **1** can be viewed as a typical superhalogen anion and is possible to be synthesized and characterized in future experiments.

## 1. Introduction

The non-classical bonding of atoms constantly refreshes our understanding and promotes the development of chemistry and materials science. Planar tetracoordinate carbon (ptC) was first introduced as a transition state by Monkhorst in 1968 [1]. Compared with typical tetrahedral structures, in terms of energies, most ptC species are usually unstable with respect to their tetrahedral carbon (thC) structures.

Is it possible to stabilize ptC using new strategies? Based on an in-depth bonding analysis of hypothetical planar CH_4_, Hoffmann and coworkers creatively put forward the “electronic” and “machanical” strategies to stabilize ptC species in 1970, which represents a milestone work in the field [2]. Using the “electronic” strategy, Schleyer predicted the first viable molecule ptC structure, 1,1-dilithiocyclopropane, in 1976 [3], noting it is only a local minimum on the potential energy surface. Since then, through the tireless efforts of the theoretical and experimental researchers, an array of ptC, planar pentacoordinate carbon (ppC), and planar hexacoordinate carbon (p6C) species were computationally designed, spectrographically characterized, and experimentally synthesized in the last half-century [4,5,6,7]. Among these, the successful examples include CAl_4_^2−^ based ptC, ptC CAl_11_^−^ molecular rotor, CAl_5_^+^ based ppC clusters, and p6C CE_3_M_3_^+^ (E = S–Te and M = Li–Cs) clusters [8,9,10,11,12,13,14,15]. Note that the CAl_4_^2−^ dianion is isoelectronic to the CAl_2_Si_2_ cluster, which was predicted by Schleyer and Boldyev in 1991 [16]. The achievements of planar hypercoordinate carbon (phC) species arouse the interest of chemists to explore the unclassical bonding of the other main-group atoms. The ptO OAl_4_, ptN NAl_3_Si, NAl_4_^−^, and ptB BAlSi_3_ clusters were also predicted [16]. The concept of phCs is naturally extended to heavier silicon, germanium congeners such as SiAl_4_^−^, GeAl_4_^−^, SiMg_4_In^−^, SiSb_3_M_3_^+^ (M = Ca, Sr, Ba), etc. [17,18,19]. Metal atoms seem to form hypercoordination bonding more easily than non-metal atoms due to their lower electronegativity. Recently, the ppBe BeM_5_^+^ (M = Cu, Ag, Au), p6Be Be©Be_6_Cl_6_, and p6Ga GaBe_6_Au_6_^+^ were predicted as the global minima (GMs) [20,21,22]. In 2023, Cui et al. reported a series of planar pentacoordinate s-block metals, including LiNa_5,_ Li_5_Mg^+^, Na_5_Mg^+^, K_5_Ca^+^, CaRb_5_^+^, Rb_5_Sr^+^, and SrCs_5_^+^ [23]. Hydrogen is the smallest atom in the periodic table. Can it become a new member of the planar hypercoordination family? In 2024, Guha et al. reported the first ptH In_4_H^+^ GM, which initiated the phH chemistry [24]. Shortly after, they predicted ptH Li_4_H_4_^−^ and ppH Li_5_H_6_^−^ [25,26]. Planar hypercoordination bonding is being extended to the rest of the periodic table.

The classical coordination modes of halogen (X = F, Cl, Br, I) atoms in the periodic table mainly include terminal (µ^1^-X), bridging (µ^2^-X), and face-capping (µ^3^-X). The planar hypercoordination of halogen atoms has been neglected for a long time, perhaps due to their strong electronegativity. In 2021, the first series of ptF clusters, including FIn_4_^+^, FTl_4_^+^, FGaIn_3_^+^, FIn_2_Tl_2_^+^, FIn_3_Tl^+^, and FInTl_3_^+^ were predicted by Merino and coworkers [27]. Recently, the alkali metal decorated ptF H_3_Li_4_F^−^ doublet cluster with *C*_2*v*_ symmetry was reported [28]. As the next heavier congener, chlorine possesses similar bonding properties to the most electronegative fluorine atom. In 2002, Li et al. investigated the structure and thermodynamic properties of the ptCl ClF_4_^−^, which possesses a perfect square structure [29]. As far as we all know, the ppCl GM cluster has not yet been reported in the literature up to now. Is it possible to extend the concept of planar pentacoordination to chlorine atoms? The answer appears to be “yes”. Increasing the composition of the ionic bonding between the ligand and chlorine atom is an effective strategy. Stimulated by the design of p6C CE_3_M_3_^+^ (E = S–Te and M = Li–Cs) clusters, we choose to use alkali metal Li as the ligand atom to stabilize phCl. Herein, we report a star-like *D*_5*h*_ cluster **1**, which represents the first ppCl GM species.

In 1981, Gutsev and Boldyrev put forward the concept of “superhalogen”, which refers to a series of unique species with electron affinity higher than chloride (3.61 eV) [30]. They also proposed a formula MX_k+1_ to design superhalogens, where M is a central atom, X is electronegative atoms as ligands such as F, Cl, Br, I, O, H, etc, and k is the valence of M. They obtained some examples of typical superhalogens such as BO_2_, AlO_2_, BeF_3_, NO_3_, PO_3_, ClO_4_, BF_4_, SiF_5_, PF_6_, etc., using the above formula. In 1999, Wang et al. characterized the MX_2_^−^ (M = Li, Na; X = Cl, Br, I) superhalogen anions by photoelectron spectroscopy [31]. Finding more electronegative ligands is an effective strategy for designing the superhalogen. As the classical electrophiles in the organic syntheses, species such as NO_2_, SHO_3_, CF_3_, CCl_3_, COOH, CHO, CONH_2_, and COOCH_3_ were used as ligands to design new superhalogen anions [32]. Subsequently, Anusiewicz reported new superhalogen anions using the acidic functional groups (ClO_4_, ClO_3_, ClO_2_, ClO, NO_3_, PO_3_, H_2_PO_4_, HSO_4_, HCO_3_, SH) as ligands [33]. In 2022, Srivastava predicted M(BO)_k+1_^−^ anions, which are novel superhalogens based on boronyl ligands [34]. Since the concept of superhalogen was proposed, a large number of superhalogens with novel structures have been theoretically designed and characterized spectrographically [35]. Although planar hypercoordinate chemistry and superhalogens are the hotspots of chemistry and materials science, they are not related to each other. Is it possible to design superhalogen containing planar hypercoordinate atoms? Such species must be exciting. Recently, we reported a square-like *D*_4*h*_ H©K_4_H_4_^−^ anion with one planar tetracoordinate hydrogen (ptH) center. Interestingly, the calculated ground-state vertical electron detachment energy (VDE) of H©K_4_H_4_^−^ is 3.51 eV at the CCSD(T) level, which can be seen as a pseudo-halogen anion [36]. Whether the ppCl **1** has a higher VDE? Excitingly, **1** has the ground-state VDE 7.87 eV at the CCSD(T) level, which is much larger than the electron affinity of Cl (3.61 eV). Thus, ppCl **1** can be viewed as a superhalogen anion. Therefore, the present results not only predict the first ppCl system but also build an interesting link between ppCl and superhalogens.

## 2. Computational Details

An unbiased exploration of the potential energy surfaces of Li_5_Cl_6_^−^ was carried out using the Coalescence Kick (CK) algorithm, initially at the PBE0/def2-SVP level [37,38]. We integrate the CK program and Gaussian 16 program effectively by compiling the two [39]. About 6000 initial structures were probed (3000 singlets and 3000 triplets). The top twenty low-lying structures were then fully reoptimized at the PBE0-D3(BJ)/def2-TZVPP level, and relative energies were calculated at the CCSD(T)/def2-TZVPP, including the zero-point energy correction at the PBE0-D3(BJ)/def2-TZVPP level [40,41]. Thus, the energy discussion is based on the CCSD(T)/def2-TZVPP/PBE0-D3(BJ)/def2-TZVPP results. The T1 diagnostic value is an important criterion for determining whether a species has a multi-reference character to be considered. If T1 diagnostic values <0.02 of the converged CCSD wavefunction, suggesting that the single-reference character method is suitable. All T1 diagnostic values of the converged CCSD wavefunction are lower than 0.02, suggesting that the multi-reference character of the low-lying isomers of Li_5_Cl_6_^−^ can be neglected. The Born–Oppenheimer molecular dynamics (BOMD) simulations were performed at PBE0/def2-TZVP level to characterize dynamic stability [42]. We used the optimized structure at the PBE0/def2-TZVP level as the initial structure of the BOMD simulation. The Maxwell distribution produces the initial velocity at a set temperature. Natural bond orbital (NBO) analysis was carried out to obtain Wiberg bond indices (WBIs) and natural population analysis (NPA) atomic charges [43]. The canonical molecular orbital (CMO), adaptive nature density partitioning (AdNDP), electron localization function (ELF), and quantum theory atoms in molecules (QTAIM) analyses were performed to gain insight into bonding [44,45,46]. Orbital composition analysis helps us to deeply understand the nature of chemical bonding, which orbital compositions were performed using Multiwfn [47]. All electronic structure calculations were done with Gaussian 16. Magnetically induced current density was calculated using the GIMIC program to facilitate the analysis of electron delocalization [48,49,50]. The interacting quantum atoms (IQA) analyses was performed using the ADF (2023) program [51]. Molecular structures, CMOs, and AdNDP results were visualized using the CYLview, GaussView, and Molekel programs, respectively [52,53].

## 3. Result and Discussion

### 3.1. Structures and Stability

**Figure 1:**
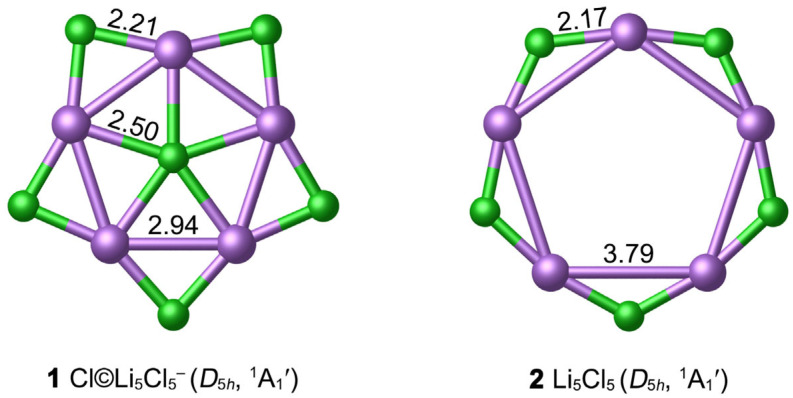
Optimized structures of **1** and **2** clusters at the PBE0-D3(BJ)/def2-TZVPP level. The bond distances are shown in Å.

The optimized GM structure **1** at the PBE0-D3(BJ)/def2-TZVPP level is depicted in Figure 1, in comparison with the hollow Li_5_Cl_5_ (**2**) star. Table S1 listed the lowest vibrational frequencies of ppCl *D*_5*h*_ **1** at nine classical theoretical levels, which are in the range of 17.2 to 23.0 cm*^−^*^1^, revealing that **1** is a true minimum structure. As shown in Figure 1, **1** possesses a star-like structure with the *D*_5*h*_ symmetry, which is composed of one Cl center, Li_5_ ring and five Cl bridges at the periphery. Geometrically, the center Cl atom is well matched with the outer Li_5_Cl_5_ star so that the **1** molecule possesses a perfect planar structure. It should be noted that the central ppCl atom has strict requirements on the geometry of the ring formed by the ligands. According to our calculations, F©Li_5_F_5_^−^ (*D*_5*h*_, ^1^A_1_′) is only a second-order saddle point on the potential energy surfaces, with two degenerate imaginary frequencies (17.4i cm*^−^*^1^) at the PBE0-D3(BJ)/def2-TZVPP level. 

**Figure 2:**
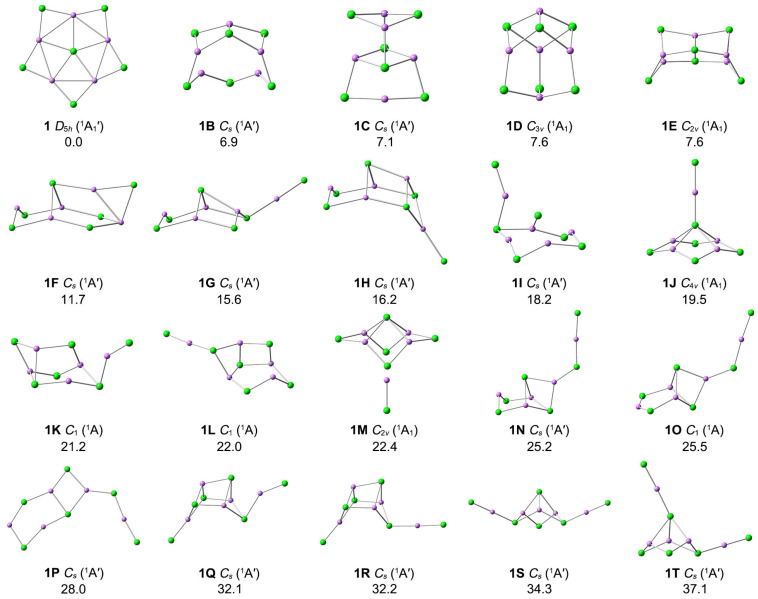
Optimized structures for the top 20 low-lying isomers of Li_5_Cl_6_^−^ at the PBE0-D3(BJ)/def2-TZVPP level. The relative energies are listed in kcal mol*^−^*^1^ at the single-point CCSD(T)/def2-TZVPP//PBE0-D3(BJ)/def2-TZVPP levels, with zero-point energy (ZPE) corrections at PBE0-D3(BJ)/def2-TZVPP.

The optimized top twenty low-lying structures at the PBE0-D3(BJ)/def2-TZVPP level are shown in Figure 2, and relative energies were calculated at the CCSD(T)/def2-TZVPP level. The optimized Cartesian coordinates for the low-lying isomers are given in Supporting Information. **1** is the true GM on the potential energy surfaces of Li_5_Cl_6_^−^, being 6.9 kcal mol*^−^*^1^ more stable than the most competitive isomer (**1B**) at the single-point CCSD(T)/def2-TZVPP//PBE0-D3(BJ)/def2-TZVPP level. Both Cl and Li atoms are mainly bonding with each other by di-coordination and tri-coordination in **1B**–**1T** isomers. The **1J** structure is obtained when one of the peripheral Li–Cl units in structure **1** moves above and bonds with the central chlorine atom, which is 19.5 kcal mol*^−^*^1^ higher than the GM at the CCSD(T) level. **1M** is obtained by moving down the Li–Cl unit bonded with the central Cl atom in **1J**. **1B**–**1T** low-lying isomers are three-dimensional structures, except for **1P**. The triplet state isomers are anticipated to be higher in energy with respect to their corresponding singlets. Indeed, the most stable triplet structure is 120.5 kcal mol*^−^*^1^ higher than **1**. In addition, to examine the effect of the diffusion function, we reoptimized the structure of **1** at PBE0-D3(BJ)/def2-TZVPPD level. The vibration frequencies are also calculated at the same level. As shown in Table S2, the addition of the diffusion function has little effect on the system. Thus, the discussion following this article is mainly based on the PBE0-D3(BJ)/def2-TZVPP results.

As depicted in Figure 1, the bond distance between the Li atom and the center Cl in **1** is 2.50 Å, which is 0.18 Å longer than the recommended covalent Li–Cl single bond length (2.32 Å) [54]. However, it is much shorter than the van der Waals bond length (3.94 Å); correspondingly, the WBI values of Li–Cl are small (0.05), so the Li–Cl bonding is largely ionic due to the electronegativity difference between the Li and Cl. There is partially covalent bonding between the Li atom and the peripheral Cl atoms, according to the bond distance (2.21 Å) and WBI (0.13). The structure of the periphery Li_5_Cl_5_ ring seems to be relatively rigid, which is beneficial to maintain the planar structure of the whole **1** system. The interatomic distance of Li–Li in **1** is 2.94 Å, exceeding the sum of covalent radii of two Li atoms (2.66 Å). The small WBI_Li–Li_ (0.001) value indicates that there is no covalent bonding between them. Note that the bond distance between the central Cl and the outer Cl atom is 3.67 Å, which is close to the van der Waals bond length (3.64 Å).

**Figure 3:**
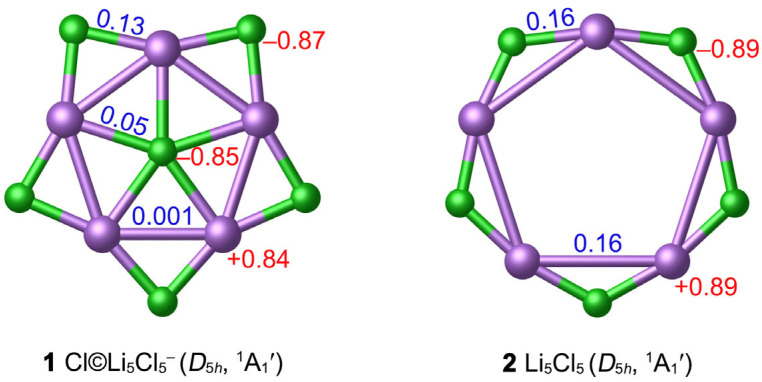
Calculated WBIs (blue color) and NPA charges (in ∣e∣, red color) of **1** and **2** at the PBE0-D3(BJ)/def2-TZVPP level.

The considerable difference between Cl (3.16) and Li (0.98) in the atomic electronegativity determines the NPA charge distribution of **1**. There are substantial charge transfers from Li to Cl. As depicted in Figure 3, the central Cl atom carries a negative charge of −0.85 ∣e∣, which is close to the peripheral Cl (−0.87 ∣e∣) in **1**. Each Li atom carries a significantly positive charge (+0.84 ∣e∣). This negative-positive-negative charge distribution from the center to the periphery is conducive to the stability of the system via Coulomb attractions. The ppCl cluster is indeed a planar “core–shell” system with relatively separated inner Cl^−^ and outer Li_5_Cl_5_ rings. According to NPA charge data, **1** should be formulated as [Cl]^−^©[Li_5_Cl_5_].

The hollow *D*_5*h*_ **2** star is a true local minimum (LM) at the PBE0-D3(BJ)/def2-TZVPP level. The Li–Li bond distance (3.79 Å) is longer than the van der Waals bond length. Introducing one Cl^−^ anion into the center of Li_5_Cl_5_ can effectively shrink the Li_5_ ring and enhance the stability of the system. To further examine the thermodynamic stability of the ppCl **1** star, we calculated the “insertion energy” of the Cl^−^ center (∆E) in Li_5_Cl_5_ + Cl^−^ = Cl©Li_5_Cl_5_^−^ process. With zero-point energy corrections included, the negative energy changed −90.38 kcal mol*^−^*^1^ at the PBE0-D3(BJ)/def2-TZVPP level, indicating that the insertion of ppCl^−^ to form the ppCl **1** star is favored thermodynamically. In addition, the energy change ∆E of the reaction 6Cl^−^ + 5Li^+^ = Cl©Li_5_Cl_5_^−^ is −1066.11 kcal mol*^−^*^1^, further supporting the stability of **1**.

**Figure 4:**
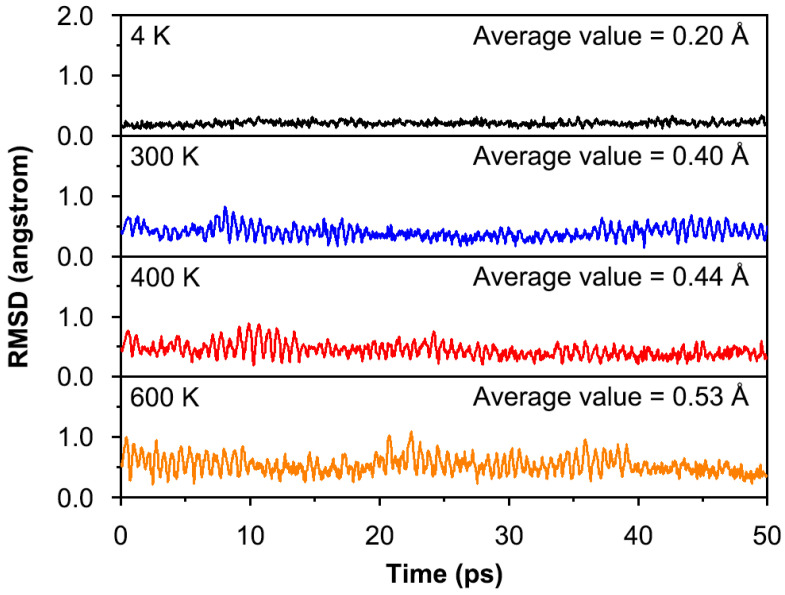
Calculated RMSDs of **1** during the BOMD simulations for 50 ps at PBE0/def2-TZVP level at temperatures of 4, 300, 400, and 600 K.

For one cluster, dynamic stability is also important for experimental observation. To validate the dynamic stability of **1**, BOMD simulations were performed for 50 ps at PBE0/def2-TZVP level at temperatures of 4, 300, 400, and 600 K. The evolution of the structure during the simulation was evaluated by the root-mean-square deviations (RMSDs) of the atomic position of each frame relative to the optimized structure at the PBE0/def2-TZVP level. The calculated corresponding average RMSDs are 0.20, 0.40, 0.44, and 0.53 Å, respectively, as shown in Figure 4. It is easy to understand that the average RMSD values increase following the rising of simulated temperatures. In general, the fluctuations in the RMSD plot were not significant. The BOMD results indicate that the structure of **1** is basically maintained during the simulations, which is dynamically stable below 600 K against isomerization and decomposition. Minor spikes are due to movements of the ppCl atom up and down the Li_5_ plane. The energy difference (6.9 kcal mol*^−^*^1^) between **1B** and GM seems to be not large. Can the GM structure transform into the **1B** structure at 600 K? To answer this question, a 10 ps BOMD simulation was performed starting from the **1B** structure at 600 K, as shown in Figure S1. After a brief period of a few picoseconds, structure **1B** is transformed into a lower energy structure **1**. In other words, **1** is still more stable than **1B** at 600 K. Overall, although the structure of highly ionizing **1** has some flexibility, it is still dynamically stable.

### 3.2. Chemical Bonding

What is it that makes cluster **1** such a perfect planar structure? Ionic bonding alone does not seem to guarantee the planar structure of **1**, although it dominates the stability of the system. The planarization of **1** may be related to multicenter bonds. To elucidate the bonding characteristics of the exotic ppCl cluster, we performed the AdNDP analysis. The program was developed by Zubarev and Boldyrev, and they found that the bonding scheme is insensitive to different basis sets [43]. AdNDP analysis recovers not only the typical Lewis bonding elements, including lone pairs (LPs) and 2c-2e bonds, but also delocalized *n*c-2e (n ≥ 3) bonds. As the extended version of NBO analysis, the AdNDP analysis is intuitive and essential. **1** is a 48-valence-electron (48 ve) system. 

**Figure 5:**
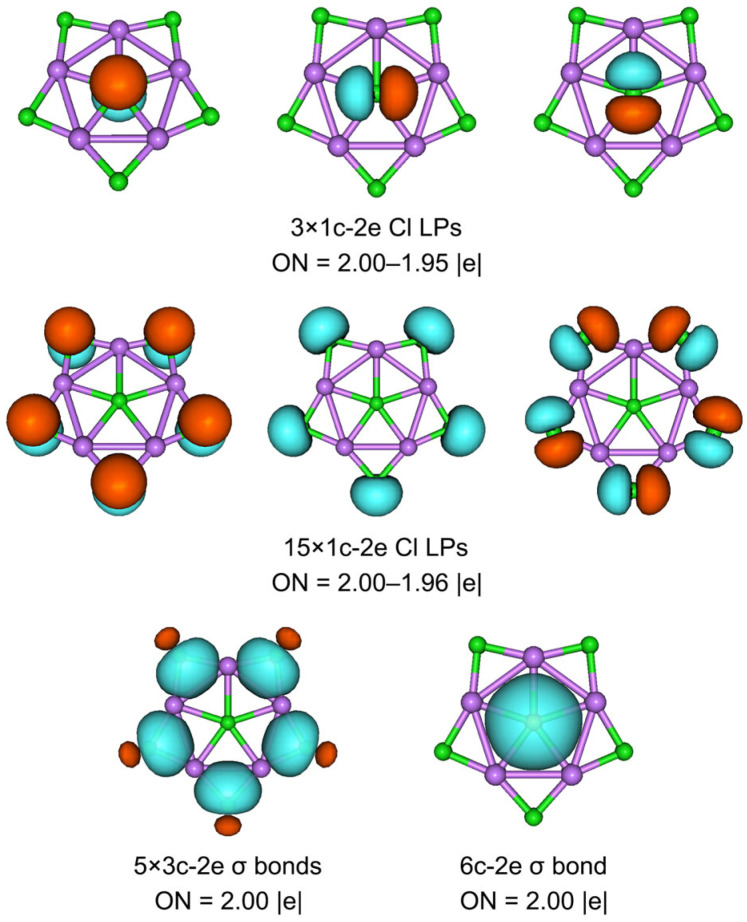
The AdNDP bonding pattern of **1**. Occupation numbers (ONs, in ∣e∣) are shown. LP stands for a lone pair.

**Figure 6:**
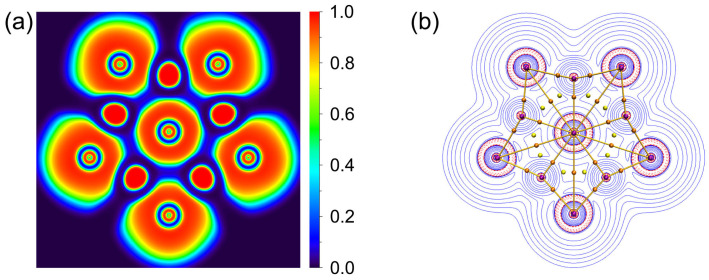
(**a**) 2D plot of ELF of **1**. (**b**) Plot of the Laplacian of electron density, bond paths and critical points. The red dashed lines denote the areas of charge concentration (∇^2^*ρ*(r) < 0), and the blue area is vice versa. The brown sticks between the atoms represent bond paths. The brown and yellow dots are bond and ring critical points, respectively.

As depicted in Figure 5, there are 18 LPs of six Cl atoms with occupation numbers (ONs) from 2.00 to 1.95 ∣e∣. The peripheral Li–Cl bonding is described as five 3c-2e Li–Cl–Li σ bonds with the ONs 2.00 ∣e∣, which hold together the Li_5_Cl_5_ star. The Li–Cl–Li σ clouds in **1** appear to be mainly located on the Cl atoms, which is because Cl is more electronegative than Li. The ppCl center is stabilized via one delocalized 6c-2e σ bond with the Li_5_ ring around it (ON 2.00 ∣e∣). If the 6c-2e bond is approximated as the lone pair of Cl, its ON value reaches 1.83 ∣e∣. Such a small ON value difference implies a low orbital overlap between the ppCl and the Li_5_ ring. Thus, the chlorine atom in the center is decisive for this 6c-2e bond, which indicates that the electrostatic interaction between the ppCl and the Li_5_ ring is dominated, whereas the covalent interaction is reflected in the multicenter bond with almost no aromatic properties. As shown in Figure 6a, the ELF diagram reveals that the electron pairs are mainly concentrated around the Cl atoms, consistent with one central 6c-2e and five outer 3c-2e σ bonds. The contour plot of the Laplacian distribution of the charge density of **1,** along with the bond critical points and ring critical points, is shown in Figure 6b. The clear bond critical points further support that the central Cl atom is a true ppCl. It also indicates the ionic multicenter bonding nature between Cl and Li atoms. In addition, no bond paths and bond critical points were found between the Li–Li bonds, which is consistent with the small WBI_Li–Li_. According to the AIM analysis, as shown in Figure S2, the central and each peripheral Cl carry the negative charges −0.92 ∣e∣ and −0.91 ∣e∣, respectively, while each surrounding lithium atom carries a positive charge +0.89 ∣e∣. The interactions between the central Cl and Li atoms are dominated by electrostatic interactions supported by the low value of the delocalization index (0.05).

**Figure 7:**
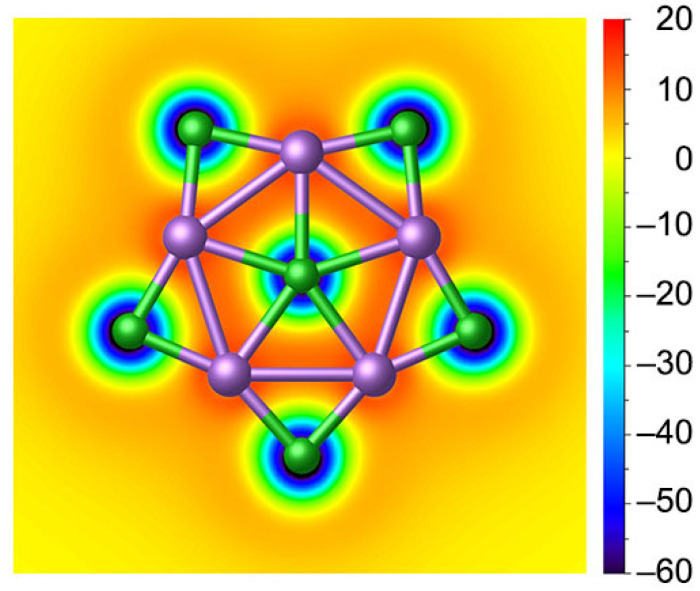
Color-filled map of NICS(0)_zz_ for **1**. Negative values indicate aromaticity. The 0 in parentheses represents the height above the molecular plane (in Å).

From a qualitative perspective, five delocalized 3c-2e Li–Cl–Li σ bonds along the periphery match the (4*n* + 2) Hückel rule with *n* = 2, making the planar complex σ-aromatic in nature. Nucleus-independent chemical shifts in the z-direction, NICS_zz_, represent one good criterion for aromaticity [55]. To intuitively probe the aromaticity, the color-filled map of NICS(0)_zz_ of **1** is plotted in Figure 7. The chemical shielding areas with negative NICS(0)_zz_ values are mainly distributed around the Cl monoanion with lone pair electrons; such delocalization is more like the delocalization of Cl^−^ anion and slightly extended towards Li but failed to cover the Li_5_©Cl ring since the 6c-2e σ bond is mainly the contribution of LP of Cl^−^. High ionization weakens the contribution of aromaticity to the stability of the system. In other words, the stability of ppCl in **1** is mainly dictated by the ionic bonding and multicentre bonding.

Current density computations were conducted using the GIMIC program at the PBE0-D3(BJ)/def2-TZVPP level, with an external magnetic field perpendicular to the **1** ring. The ring current strength was determined by integrating the ring current in planes perpendicular to the molecular planes.

Figure S3a displays a vector representation of the current, indicating a significant influence on the nucleus with no obvious circulation. Figure S3b provides schematic representations of the identified ring current circuits and their strengths in nA/T. It clearly shows that **1** exhibits certain loops only around the Cl atoms, with strengths of 8.4 and 8.7 nA/T. It is worth noting that the region with a ring current circuit was localized around ppCl and slightly extended towards Li but failed to cover the Li_5_@Cl loop. Figure S3c illustrates the integration plane, with the direction of integration following the direction of the arrow. Figure S3d presents current strength profiles for the integration plane. From left to right, the first peak corresponds to the contribution of the Li atoms, the second peak to the central Cl atom, and the third peak to the peripheral Cl atoms.

IQA provides a more accurate description of the type of interatomic interactions. To gain an in-depth understanding of the nature of ionic bonding, the IQA analyses were performed for the **1** anion and hollow **2** stars. In simple terms, the interatomic interaction energy ($V_{IQA}^{int}$) can be decomposed into electrostatic interaction energy ($V_{C}^{int}$) and covalent interaction energy ($V_{XC}^{int}$). As listed in Table 1, the $V_{C}^{int}$ (Cl–Li) is −105.5 kcal mol*^−^*^1^ for **1**, which is much larger than the covalent interaction $V_{XC}^{int}$ (Cl–Li) term. Thus, the interaction between the central Cl atom and the ligand Li is basically ionic bonding. The coordination number in a chemical species refers to “the number of other atoms directly linked to that specified atom”, according to the definition given by IUPAC. So, the central Cl atom of **1** is one true ppCl. The covalent component of the interaction between the outer Cl and Li is slightly larger than that of the central Cl atom. However, it is still dominated by ionic bonding. As expected, the interaction between two Li atoms is electrostatic repulsion. It should be noted that the absolute value of $V_{IQA}^{int}$ (Cl^a^–Li) in Li_5_Cl_5_ is slightly smaller than that of **1**, indicating that the addition of Cl^−^ anion is also conducive to strengthening the interaction between outer Cl and Li atoms.

### 3.3. Superhalogen Anion Characteristic

**Figure 8:**
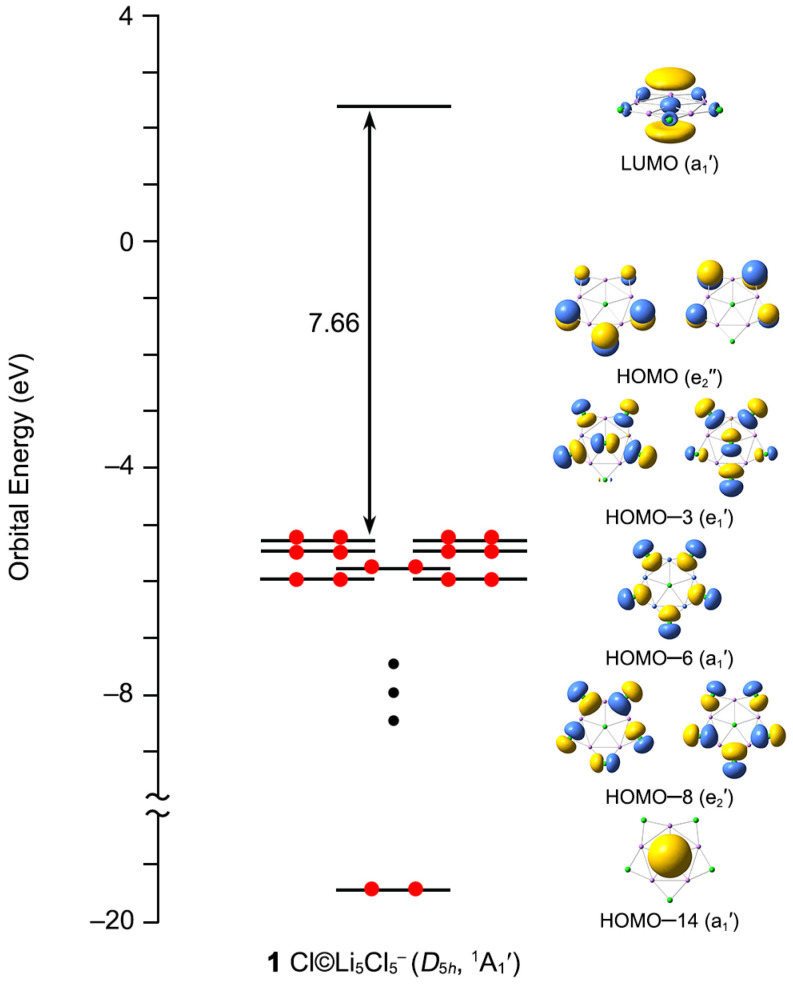
The canonical molecular orbital energy diagram of **1**.

The energy gap between the HOMO (highest occupied molecular orbital) and the LUMO (lowest unoccupied molecular orbital) can be used to characterize the electronic stabilities of the target clusters. The larger the value of the HOMO–LUMO gap, the higher the chemical stability and the lower reactivity are. As shown in Figure 8, **1** has a large HOMO–LUMO energy gap (7.66 eV) at PBE0-D3(BJ)/def2-TZVPP level, suggesting that the ppCl star is highly chemically inert. Note that the LUMO (a_1_′) is a Li-based orbital and possesses the positive orbital energy (2.38 eV). Adding one electron to its LUMO, *D*_5*h*_ Cl©Li_5_Cl_5_^2−^ is formed, which is unstable with three imaginary frequencies at the PBE0-D3(BJ)/def2-TZVPP level. As listed in Table S2 (Supporting Information), the doubly degenerate HOMO (e_2_″) is dominated by 3p_z_ compositions of five outer Cl atoms, which have a negative orbital energy (−5.28 eV). Losing an electron from the HOMO is detrimental to the stability of the system. The neutral *D*_5*h*_ Cl©Li_5_Cl_5_ is a secondary saddle point structure at the PBE0-D3(BJ)/def2-TZVPP level. Thus, the 48 ve counting is ideal for **1** star and adding or removing an electron can deteriorate its electronic stability. HOMO–3 (e_1_′), HOMO–6 (a_1_′), and HOMO–8 (e_2_′) are corresponding to the five 3c-2e Li–Cl–Li σ bonds at the periphery. Meanwhile, HOMO–14 (a_1_′) is responsible for the central 6c-2e σ bond, where it is dominated by Cl s atomic orbital (AO) (90.50%; Table S2). The electron cloud is clearly centered on the central Cl atom, which is consistent with the results of the AdNDP analysis.

For an anion, a large HOMO–LUMO energy gap usually means that it is chemically inert. The calculated ground-state VDE of **1** is 7.87 eV at the CCSD(T)/def2-TZVPP//PBE0-D3(BJ)/def2-TZVPP level. According to the definition of superhalogen anions, **1** is, therefore, a true superhalogen anion. Most of the reported superhalogen anions possess three-dimensional structures, and almost no planar star structure has been found. As the first ppCl GM cluster with the superhalogen anion character, **1** builds an important link between the superhalogen and planar hypercoordination chemistry.

## 4. Conclusions

In summary, the first star-like ppCl GM **1** cluster has been successfully predicted in this work by increasing the composition of the ionic bonding between the central atom and the ligands. Cluster **1** possesses good thermodynamic and kinetic stability, which makes it a viable candidate for future experimental synthesis. Chemical bonding analysis indicates that the ionic multicenter bonds between Cl and Li maintain the stability of the **1** system. Strikingly, it is an exotic Cl-based superhalogen anion with large VDE (7.87 eV) at CCSD(T) level. The merge of ppCl and superhalogen anion character is an amazing thing. One superhalogen anion containing a ppCl center or ppCl species with the superhalogen character was unknown in the literature. The current results will motivate further theoretical and experimental studies on novel ppCl complexes as well as superhalogen species.

## Figures and Tables

**Table 1:** Energy components of IQA for the **1** and **2** systems at the PBE0/TZ2P level; $V_{IQA}^{int}$, $V_{C}^{int}$, and $V_{XC}^{int}$ are the interatomic IQA interaction energy, the Coulombic, and exchange-correlation energy components, respectively, in kcal mol^−1^.

Title 1	1	2
$V_{IQA}^{int}$ (Cl–Li)	−110.7	—
$V_{C}^{int}$ (Cl–Li)	−105.5 (95.3%)	—
$V_{XC}^{int}$ (Cl–Li)	−5.2 (4.7%)	—
$V_{IQA}^{int}$ (Li–Li)	89.2	69.3
$V_{C}^{int}$ (Li–Li)	89.2 (100.0%)	69.3 (100.0%)
$V_{XC}^{int}$ (Li–Li)	0.0 (0.0%)	0.0 (0.0%)
$V_{IQA}^{int}$ (Cl ^a^–Li)	−139.4	−136.0
$V_{C}^{int}$ (Cl ^a^–Li)	−124.9 (89.6%)	−120.1 (88.3%)
$V_{XC}^{int}$ (Cl ^a^−Li)	−14.5 (10.4%)	−15.9 (11.7%)

^a^ The Cl atom at the periphery.

## Data Availability

Data are contained within this article and Supplementary Materials.

## Supplementary Materials

The following supporting information can be downloaded at: https://www.mdpi.com/article/10.3390/molecules29163831/s1, Table S1: The lowest vibrational frequency at nine classical theoretical levels for the global-minimum structure **1** (*D*_5*h*_, ^1^A_1_′); Table S2: Bond lengths (*r*, Å) and the lowest vibrational frequency (*v*_min_, cm^−1^) of the **1** computed at the PBE0-D3(BJ)/def2-TZVPP and PBE0-D3(BJ)/def2-TZVPPD level; Table S3: Composition analysis of canonical molecular orbitals (CMOs) for the GM (**1**) structure at the PBE0/def2-TZVPP level; Figure S1: Calculated RMSDs of **1B** during the BOMD simulation for 10 ps at PBE0/def2-TZVP level, at the temperature of 600 K. The structure given on the graph is obtained from the reoptimization as well as after eliminating the imaginary frequency at PBE0/def2-TZVP level; Figure S2: Calculated delocalization index (blue color), and QTAIM atom charges (in |e|, red color) of **1** at the PBE0-D3(BJ)/def2-TZVPP level; Figure S3: a) Vector plots of current density at 0.0 Å. b) schematic of identified ring currents with their strengths in nA/T. c) Selected integration plane. d) Current strength profiles for the integration plane; Cartesian coordinates of top 20 low-lying isomers of Li_5_Cl_6_^−^ at PBE0-D3(BJ)/def2-TZVPP.
