# Supplementary material for: Cl©Li5Cl5−: A Star-like Superhalogen Anion Featuring a Planar Pentacoordinate Chlorine at the Center
Source: Molecules. 2024 Aug 12;29(16):3831. doi: 10.3390/molecules29163831 (PMC11357008; doi:10.3390/molecules29163831)
Supplement: Supplementary file 1 [file molecules-29-03831-s001.zip › molecules-3121835-supplementary.pdf]

SUPPLEMENTARY MATERIALS

Cl© $Li_5Cl_5^-$: A Star-like Superhalogen Anion Featuring a Planar Pentacoordinate Chlorine at the Center

Li-Xia Bai, Cai-Yue Gao, Jin-Chang Guo * and Si-Dian Li *

Key Laboratory of Materials for Energy Conversion and Storage of Shanxi Province,

Institute of Molecular Science Shanxi University Taiyuan 030006, China

E-mail: guojc@sxu.edu.cn; lisidian@sxu.edu.cn

Supplementary Materials

Table S1. The lowest vibrational frequency at nine classical theoretical levels for the global-minimum structure **1** (D_{5h}, $^1A_1'$).

Table S2. Bond lengths (r, Å) and the lowest vibrational frequency (ν_{min}, cm^{-1}) of the **1** computed at the PBE0-D3(BJ)/def2-TZVPP and PBE0-D3(BJ)/def2-TZVPPD level.

Table S3. Composition analysis of canonical molecular orbitals (CMOs) for the GM (**1**) structure at the PBE0/def2-TZVPP level.

Figure S1. Calculated RMSDs of **1B** during the BOMD simulation for 10 ps at PBE0/def2-TZVP level, at the temperature of 600 K. The structure given on the graph is obtained from the reoptimization as well as after eliminating the imaginary frequency at PBE0/def2-TZVP level.

Figure S2. Calculated delocalization index (blue color), and QTAIM atom charges (in |e|, red color) of **1** at the PBE0-D3(BJ)/def2-TZVPP level.

Figure S3. a) Vector plots of current density at 0.0 Å. b) schematic of identified ring currents with their strengths in nA/T. c) Selected integration plane. d) Current strength

profiles for the integration plane.

Cartesian coordinates of top 20 low-lying isomers of $Li_5Cl_6^-$ at PBE0-D3(BJ)/def2-TZVPP.

Table S1. The lowest vibrational frequency at nine classical theoretical levels for the global-minimum structure **1** (D_{5h}, $^1A_1'$).

	Theoretical level	Lowest vibrational frequency (cm^{-1})
1	PBE0-D3(BJ)/def2-TZVPP	21.0
2	BP86-D3(BJ)/def2-TZVPP	19.3
3	B3LYP-D3(BJ)/def2-TZVPP	21.3
4	B3PW91-D3(BJ)/def2-TZVPP	20.0
5	TPSS-D3(BJ)/def2-TZVPP	20.9
6	MP2/def2-TZVPP	21.9
7	ωB97X-D/def2-TZVPP	22.7
8	TPSSh/def2-TZVPP	23.0
9	M06-2x/def2-TZVPP	17.2

Table S2. Bond lengths (r, Å) and the lowest vibrational frequency (ν_{min}, cm^{-1}) of the **1** computed at the PBE0-D3(BJ)/def2-TZVPP and PBE0-D3(BJ)/def2-TZVPPD level.

	PBE0-D3(BJ)/def2-TZVPP	PBE0-D3(BJ)/def2-TZVPPD
ν_{min}	21.0	18.4
$r_{Cl–Li}$	2.50	2.50
$r_{Li–Li}$	2.94	2.94
$r_{Cl^a–Li}$	2.21	2.21

[a] The Cl atom at the periphery.

Table S3. Composition analysis of canonical molecular orbitals (CMOs) for the GM (**1**) structure at the PBE0/def2-TZVPP level. (Cl^a represents the Cl atom at the periphery.)

CMO	Cl (%)	Cl^a_5 (%)	Li_5 (%)	
	s/p	s/p/d	s/p	total
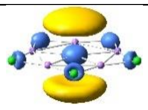 LUMO (a_1')	0.83/0.00	0.00/0.00/2.90	**96.05**/0.00	**99.78**
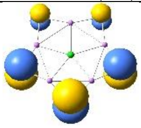 HOMO (e_2'')	0.00/0.00	0.00/**99.80**/0.00	0.00/0.00	**99.80**
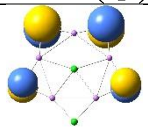 HOMO′ (e_2'')	0.00/0.00	0.00/**99.80**/0.00	0.00/0.00	**99.80**
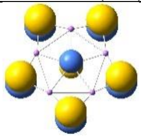 HOMO-1 (a_2'')	0.00/5.85	0.00/**91.00**/0.00	0.00/3.00	**99.85**
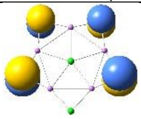 HOMO-2 (e_1'')	0.00/0.00	0.00/**96.62**/0.00	0.00/2.44	**99.06**
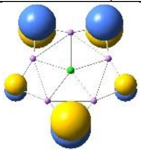 HOMO-2′ (e_1'')	0.00/0.00	0.00/**96.62**/0.00	0.00/2.44	**99.06**
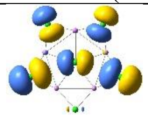 HOMO-3 (e_1')	0.00/14.96	0.00/**83.96**/0.00	0.00/0.00	**98.92**

CMO	Cl (%)	Cl^a_5 (%)	Li_5 (%)	
	s/p	s/p	s/p	total
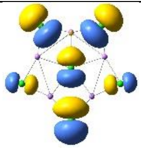 HOMO-3′ (e_1')	0.00/14.96	0.00/**83.96**/0.00	0.00/0.00	**98.92**
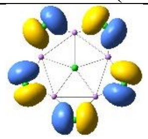 HOMO-4 (a_2')	0.00/0.00	0.00/**99.23**	0.00/0.00	**99.23**
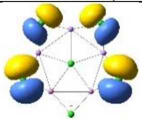 HOMO-5 (e_1')	0.00/0.53	0.00/**97.20**	1.08/0.73	**99.54**
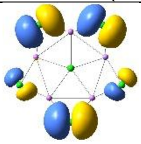 HOMO-5′ (e_1')	0.00/0.53	0.00/**97.20**	1.08/0.73	**99.54**
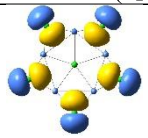 HOMO-6 (a_1')	0.55/0.00	0.00/**94.80**	2.95/0.00	**98.30**
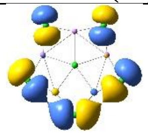 HOMO-7 (e_2')	0.00/0.00	0.00/**94.86**	2.34/0.00	**97.20**
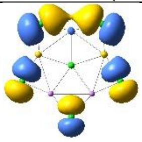 HOMO-7′ (e_2')	0.00/0.00	0.00/**94.86**	2.34/0.00	**97.20**
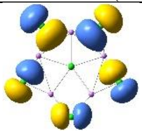 HOMO-8 (e_2')	0.00/0.00	0.00/**96.73**	1.62/0.00	**98.35**
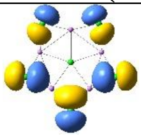 HOMO-8′ (e_2')	0.00/0.00	0.00/**96.73**	1.62/0.00	**98.35**

CMO	Cl (%)	Cl^{a}_{5} (%)	Li_5 (%)	
	s/p	s/p	s/p	total
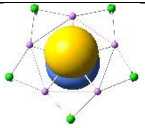 HOMO-9 (a_2'')	0.00/**91.99**	0.00/4.35	0.00/3.65	**99.99**
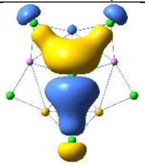 HOMO-10 (e_1')	0.00/**79.92**	0.00/13.04	2.73/2.01	**97.70**
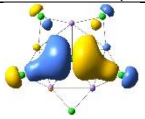 HOMO-10′ (e_1')	0.00/**79.92**	0.00/13.04	2.73/2.01	**97.70**
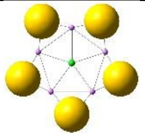 HOMO-11 (a_1')	0.88/0.00	**94.90**/0.00	3.35/0.00	**99.13**
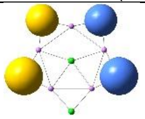 HOMO-12 (e_1')	0.00/0.00	**93.90**/0.00	1.75/2.99	**98.64**
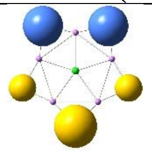 HOMO-12′ (e_1')	0.00/0.00	**93.90**/0.00	1.75/2.99	**98.64**
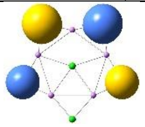 HOMO-13 (e_2')	0.00/0.00	**92.66**/0.00	0.00/6.35	**99.01**
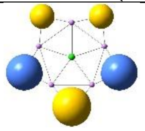 HOMO-13′ (e_2')	0.00/0.00	**92.66**/0.00	0.00/6.35	**99.01**
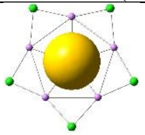 HOMO-14 (a_1')	**90.50**/0.00	0.00/0.00	0.00/6.13	**96.63**

Figure S1. Calculated RMSDs of **1B** during the BOMD simulation for 10 ps at PBE0/def2-TZVP level, at the temperature of 600 K. The structure given on the graph is obtained from the reoptimization as well as after eliminating the imaginary frequency at PBE0/def2-TZVP level.

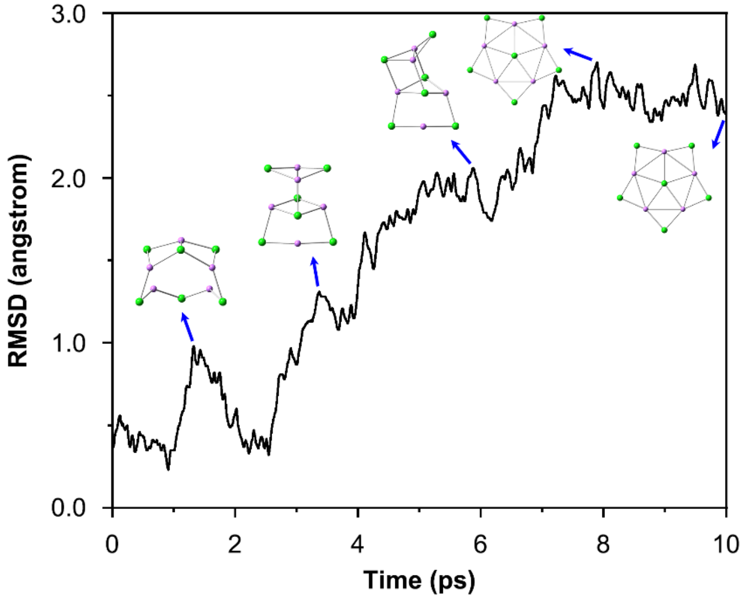

Figure S2. Calculated delocalization index (blue color), and QTAIM atom charges (in |e|, red color) of **1** at the PBE0-D3(BJ)/def2-TZVPP level.

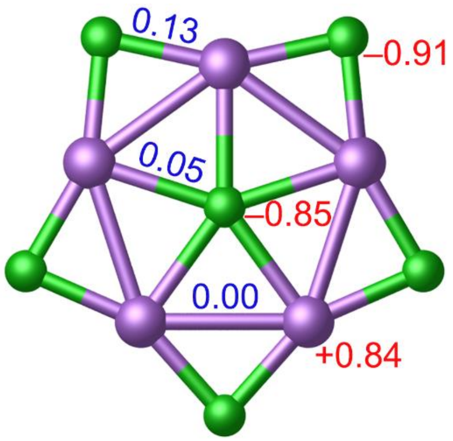

Figure S3. a) Vector plots of current density at 0.0 Å. b) schematic of identified ring currents with their strengths in nA/T. c) Selected integration plane. d) Current strength profiles for the integration plane.

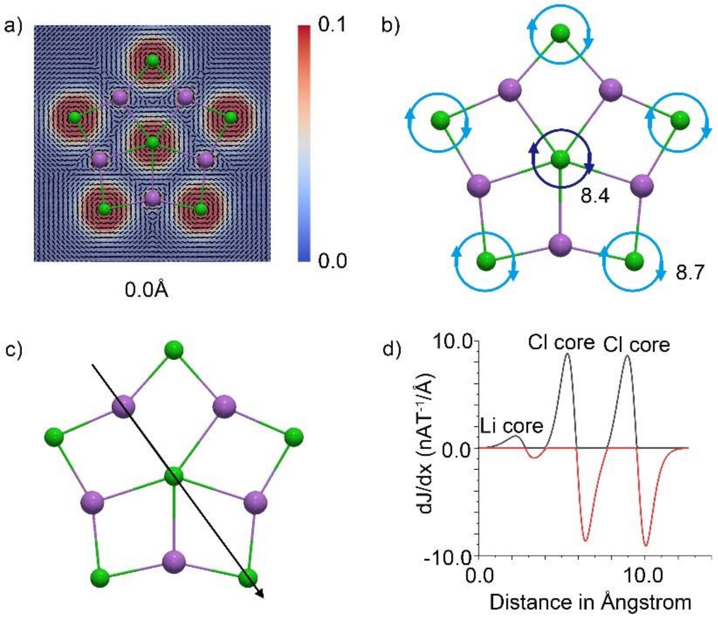

Cartesian coordinates of top 20 low-lying isomers of $Li_5Cl_6^-$ at PBE0-D3(BJ)/def2-TZVPP.

1 Cl©$Li_5Cl_5^-$ (D_{5h}, $^1A_1'$)

Li	0.00000000	2.49887500	0.00000000
Li	2.37657100	0.77219500	0.00000000
Li	1.46880200	-2.02163200	0.00000000
Li	-1.46880200	-2.02163200	0.00000000
Li	-2.37657100	0.77219500	0.00000000
Cl	0.00000000	0.00000000	0.00000000
Cl	-2.15754000	2.96959900	0.00000000
Cl	2.15754000	2.96959900	0.00000000
Cl	-3.49097300	-1.13428600	0.00000000
Cl	0.00000000	-3.67062600	0.00000000
Cl	3.49097300	-1.13428600	0.00000000

1B (C_s, $^1A'$)

Cl	0.63697806	1.54970288	2.05662000
Cl	-0.66528254	-1.71424497	2.49716400
Cl	2.56189060	-0.67471137	0.00000000
Cl	-2.69883790	0.91213603	0.00000000
Cl	-0.66528254	-1.71424497	-2.49716400
Cl	0.63697806	1.54970288	-2.05662000
Li	1.48549161	1.41071225	0.00000000
Li	1.18709548	-0.71924200	-1.86569100
Li	-1.38143190	0.27358786	1.67676300
Li	-1.38143190	0.27358786	-1.67676300
Li	1.18709548	-0.71924200	1.86569100

1C (C_s, $^1A'$)

Li	-0.41997249	2.33362074	0.00000000
Li	-1.38976053	-1.89637122	0.00000000
Li	0.14753668	0.14675751	1.60105400
Li	1.25876366	-2.11410653	0.00000000
Li	0.14753668	0.14675751	-1.60105400
Cl	-0.10090611	-2.34759454	-1.83022500
Cl	-1.79636927	0.27796750	0.00000000
Cl	0.05129181	2.34223248	-2.19917800
Cl	0.05129181	2.34223248	2.19917800
Cl	-0.10090611	-2.34759454	1.83022500
Cl	1.94075599	-0.02312421	0.00000000

1D (C_{3v}, $^1A_1$)

Cl	0.00000000	2.24823445	-1.75447467
Cl	-1.79180410	1.03449858	1.66292255
Cl	0.00000000	-2.06899716	1.66292255
Cl	1.94702815	-1.12411723	-1.75447467
Cl	1.79180410	1.03449858	1.66292255
Cl	-1.94702815	-1.12411723	-1.75447467
Li	-0.00000000	-0.00000000	-2.17859608
Li	-1.66583822	-0.96177214	0.40045823
Li	0.00000000	0.00000000	2.53360592
Li	0.00000000	1.92354429	0.40045823
Li	1.66583822	-0.96177214	0.40045823

1E (C_{2v}, $^1A_1$)

Cl	0.00000000	2.27742300	1.72182300
Cl	0.00000000	3.07792300	-1.74025300
Cl	0.00000000	-3.07792300	-1.74025300
Cl	0.00000000	-2.27742300	1.72182300
Cl	-2.13614300	0.00000000	-0.02308700
Cl	2.13614300	0.00000000	-0.02308700
Li	-1.31481000	2.10772000	-0.21526000
Li	-1.31481000	-2.10772000	-0.21526000
Li	1.31481000	2.10772000	-0.21526000
Li	0.00000000	0.00000000	1.33156600
Li	1.31481000	-2.10772000	-0.21526000

1F (C_s, $^1A'$)

Li	1.20914438	-2.73817753	0.00000000
Li	0.07015192	0.73098589	1.75796900
Li	-0.54453893	2.86052138	0.00000000
Li	-0.85880170	-1.58790038	0.00000000
Li	0.07015192	0.73098589	-1.75796900
Cl	0.95447261	-1.31601168	1.81058700
Cl	0.95447261	-1.31601168	-1.81058700
Cl	-1.57725417	0.62582681	0.00000000
Cl	0.21064504	2.93030517	2.08848300
Cl	-0.74347071	-3.85378119	0.00000000
Cl	0.21064504	2.93030517	-2.08848300

1G (C_s, $^1A'$)

Cl	0.46454508	-1.22413377	3.04864200
Cl	-1.38234828	-0.95431774	0.00000000
Cl	-0.34095358	5.97558326	0.00000000
Cl	0.02275358	-4.25489054	0.00000000
Cl	0.83477545	1.81148979	0.00000000
Cl	0.46454508	-1.22413377	-3.04864200
Li	-0.01706144	0.24236811	-1.47951000
Li	-0.28838915	-2.59931882	-1.45184600
Li	-0.28838915	-2.59931882	1.45184600
Li	-0.01706144	0.24236811	1.47951000
Li	0.25210303	3.97951707	0.00000000

1H (C_s, $^1A'$)

Li	1.87483426	0.56266725	0.00000000
Li	-0.56655250	-2.89594940	0.00000000
Li	0.26928411	-0.88388211	1.86962700
Li	-0.21337664	2.61403353	0.00000000
Li	0.26928411	-0.88388211	-1.86962700
Cl	-1.14892105	-2.55938949	2.11865700
Cl	-1.14892105	-2.55938949	-2.11865700
Cl	1.60729058	-1.69766509	0.00000000
Cl	0.83802783	1.31904640	1.82096500
Cl	-1.27376416	4.44076531	0.00000000
Cl	0.83802783	1.31904640	-1.82096500

1I (C_s, $^1A'$)

Cl	0.84684675	0.04914139	0.00000000
Cl	-0.83842722	-1.27376504	2.92081800
Cl	1.53903601	4.27747308	0.00000000
Cl	-1.96325520	1.85033203	0.00000000
Cl	1.32640045	-3.51318830	0.00000000
Cl	-0.83842722	-1.27376504	-2.92081800
Li	-1.05442632	0.34918435	1.46773900
Li	-1.05442632	0.34918435	-1.46773900
Li	0.31426047	2.57920503	0.00000000
Li	0.69280428	-1.96809988	1.45512000
Li	0.69280428	-1.96809988	-1.45512000

1J (C_{4v}, $^1A_1$)

Cl	-3.05116010	0.00000000	-1.32968218
Cl	3.05116010	0.00000000	-1.32968218

Cl	0.00000000	3.05116010	-1.32968218
Cl	0.00000000	-3.05116010	-1.32968218
Cl	0.00000000	0.00000000	4.87443382
Cl	0.00000000	0.00000000	0.53291982
Li	-1.49897162	1.49897162	-0.82496718
Li	1.49897162	-1.49897162	-0.82496718
Li	-1.49897162	-1.49897162	-0.82496718
Li	1.49897162	1.49897162	-0.82496718
Li	0.00000000	0.00000000	2.79766082

1K (C_1, ^{1}A)

Cl	3.40686200	-1.61188400	-0.72347900
Cl	-1.76639200	1.84780900	0.51227400
Cl	1.68742000	-0.23802700	2.06959800
Cl	1.86721900	2.07951900	-1.09496800
Cl	-0.58315200	-1.07828800	-0.54172200
Cl	-4.77894000	-1.01909100	-0.30779200
Li	2.86751000	0.43811100	0.03629400
Li	1.46909300	-1.76571300	0.30003200
Li	-2.87105500	-0.13370000	-0.18973600
Li	-0.35510600	0.32277800	1.40371300
Li	-0.16421000	1.25163700	-1.06246100

1L (C_1, ^{1}A)

Cl	-1.61723500	2.33785200	-0.64965600
Cl	-1.71747600	-0.65705900	1.95270600
Cl	0.01085000	-1.34773400	-1.04066800
Cl	5.67593900	-0.52142600	-0.35294000
Cl	-4.03774200	-0.83087800	-0.73895600
Cl	1.81395300	1.07252600	0.74687300
Li	0.11534900	1.01148100	-0.91629900
Li	-2.87759600	0.78124200	0.33571300
Li	3.76122600	0.20095600	0.06195700
Li	0.36028000	-0.63975300	1.13606500
Li	-2.08622700	-1.65585900	-0.14913600

1M (C_{2v}, $^1A_1$)

Cl	0.00000000	3.27278700	-0.98531900
Cl	2.02183200	-0.00000000	0.42867400
Cl	0.00000000	0.00000000	-2.48373200
Cl	0.00000000	-3.27278700	-0.98531900

Cl	-2.02183200	-0.00000000	0.42867400
Cl	0.00000000	0.00000000	4.02367900
Li	1.30456200	1.49545200	-1.08088500
Li	-1.30456200	-1.49545200	-1.08088500
Li	-1.30456200	1.49545200	-1.08088500
Li	1.30456200	-1.49545200	-1.08088500
Li	0.00000000	0.00000000	1.90581700

1N (C_s, $^1A'$)

Cl	2.85948498	1.47636094	0.00000000
Cl	6.17119191	-1.28422814	0.00000000
Cl	-0.53875808	-0.99738298	0.00000000
Cl	-3.51373115	-0.74965181	2.00637104
Cl	-3.51373115	-0.74965181	-2.00637104
Cl	-1.22878901	2.24244904	0.00000000
Li	-1.80735710	0.49446612	1.40923505
Li	0.74200196	0.97665299	0.00000000
Li	4.50683494	-0.00541810	0.00000000
Li	-1.80735710	0.49446612	-1.40923505
Li	-2.97374710	-1.57738492	0.00000000

1O (C_1, ^1A)

Cl	-3.74321800	-1.38844900	1.57524100
Cl	2.90689900	-0.88539000	-1.12033000
Cl	-1.22652600	-1.07321000	-1.55655000
Cl	6.43319500	0.64511400	0.82879300
Cl	-0.32197600	0.69849400	1.06741100
Cl	-3.66863600	2.03089600	-0.75984200
Li	-4.30589700	0.42902500	0.54624200
Li	4.66191000	-0.05799800	-0.05770700
Li	-1.69145000	-1.15327800	0.72471400
Li	0.82529000	-0.46732200	-0.60024600
Li	-1.64169700	1.09399500	-0.80977000

1P (C_s, $^1A'$)

Cl	3.77280892	-2.89498038	0.00000000
Cl	-5.25935100	-2.46077038	0.00000000
Cl	3.76480927	0.73896794	0.00000000
Cl	-3.41701874	1.43253317	0.00000000
Cl	0.30573345	3.36150608	0.00000000
Cl	0.25003516	-0.23897192	0.00000000

Li	4.92215208	-1.09385636	0.00000000
Li	1.61039633	1.62060105	0.00000000
Li	-1.22542070	1.68534111	0.00000000
Li	2.22123108	-1.23895109	0.00000000
Li	-4.22478887	-0.62341361	0.00000000

1Q (C_s, $^1A'$)

Cl	5.20286506	-0.47998111	0.00000000
Cl	1.50996113	1.51804002	0.00000000
Cl	-1.55052995	0.82914113	1.78109087
Cl	1.14603201	-1.79552296	0.00000000
Li	0.43206311	-0.13657294	-1.38629407
Li	3.10826306	-0.43802903	0.00000000
Li	0.43206311	-0.13657294	1.38629407
Li	-0.75431085	1.96951910	0.00000000
Cl	-1.55052995	0.82914113	-1.78109087
Li	-2.94258493	-0.11806882	0.00000000
Cl	-4.80625996	-1.10186275	0.00000000

1R (C_s, $^1A'$)

Cl	1.04954912	1.24534261	0.00000000
Cl	1.08966354	5.57194629	0.00000000
Cl	2.17991340	-4.12029526	0.00000000
Cl	-0.78732403	-1.78049284	1.79540000
Cl	-2.45733543	0.82728060	0.00000000
Cl	-0.78732403	-1.78049284	-1.79540000
Li	-0.61815715	0.42468900	-1.37213000
Li	1.10656325	3.49169145	0.00000000
Li	-2.12137889	-1.44170635	0.00000000
Li	-0.61815715	0.42468900	1.37213000
Li	0.62398869	-2.69133153	0.00000000

1S (C_s, $^1A'$)

Li	-1.77048500	0.40167478	1.35401800
Li	1.08477110	-0.38552084	-3.90278500
Li	0.64270406	-0.06665990	0.00000000
Li	1.08477110	-0.38552084	3.90278500
Li	-1.77048500	0.40167478	-1.35401800
Cl	2.02747303	0.15988029	5.67568600
Cl	-3.40722792	-0.23785544	0.00000000
Cl	2.02747303	0.15988029	-5.67568600

Cl	0.01463818	-0.97166998	-2.01996500
Cl	-0.54839620	1.86749694	0.00000000
Cl	0.01463818	-0.97166998	2.01996500

1T (C_s, ^{1}A′)

Li	-1.67190802	-1.96238808	0.00000000
Li	3.84848697	0.73301393	0.00000000
Li	0.37998282	-0.68331384	1.45922000
Li	0.37998282	-0.68331384	-1.45922000
Cl	-0.94076076	-2.33735754	-2.06420600
Cl	5.40698924	2.10909996	0.00000000
Cl	2.18005558	-0.76924228	0.00000000
Cl	-0.94076076	-2.33735754	2.06420600
Cl	-1.03224711	0.56657072	0.00000000
Li	-2.90528943	1.82338909	0.00000000
Cl	-4.67879181	2.90463010	0.00000000

Triplet ($C_{s,}$ ^{3}A″)

Cl	-0.46708420	0.82119841	0.00000000
Cl	2.10612220	-1.46970909	1.29948000
Cl	-4.08408224	1.24123515	0.00000000
Cl	2.67963206	2.66323873	0.00000000
Cl	2.10612220	-1.46970909	-1.29948000
Cl	-1.92841348	-2.52637763	0.00000000
Li	0.07180024	-1.59393303	0.00000000
Li	1.97881075	0.56760061	0.00000000
Li	-2.61411428	-0.41929319	0.00000000
Li	0.51750390	3.11449696	0.00000000
Li	-2.29034769	2.52516192	0.00000000
